# Impact of Secondary Prevention on Mortality in the Building Trades National Medical Screening Program: Effectiveness of Occupational High‐Risk Management

**DOI:** 10.1002/ajim.70052

**Published:** 2026-01-06

**Authors:** Knut Ringen, John M. Dement, Marianne Cloeren, Sammy Almashat, William Grier, Stella Hines, Laura S. Welch, Kim Cranford, Scott Haas, Patricia Quinn, Anna Chen, Miles Fisher

**Affiliations:** ^1^ CPWR—The Center for Construction Research and Training Silver Spring Maryland USA; ^2^ Division of Occupational and Environmental Medicine Duke University Medical Center Durham North Carolina USA; ^3^ Division of Occupational and Environmental Medicine, University of Maryland School of Medicine Baltimore Maryland USA; ^4^ Division of Pulmonary and Critical Care Medicine, School of Medicine University of Maryland Baltimore Maryland USA; ^5^ Zenith American Solutions Seattle Washington USA

**Keywords:** mortality, occupational health screening, occupational medical exams, secondary prevention

## Abstract

**Background:**

Since 1997 the Building Trades National Medical Screening Program (BTMed) has offered medical exams to construction workers employed in US nuclear weapons facilities. The process consists of two steps: (1) a detailed work history interview; and (2) a medical exam. Some participants only completed the work history interview, and we compared their mortality experience to those who also completed medical exams.

**Methods:**

We compared the mortality of 3470 work‐history‐only participants to 23,452 participants who completed both the work history interview and medical exams and, of these, 1720 who additionally participated in lung cancer screening. We used Cox proportional hazard and Poisson regression models to estimate hazard ratios and risk ratios while controlling for potential confounders.

**Results:**

Medical exam participants experienced a reduction in mortality risk of 28% for all causes combined; 27% for all respiratory diseases combined; 37% for chronic obstructive pulmonary disease; 30% for cardiovascular diseases combined; 32% for all cancers combined; 36% for lung cancer; and 53% for colorectal cancer. The more medical exams they undertook the greater the mortality risk reduction (25%, 29%, and 43% for one, two, and three medical exams, respectively), demonstrating a clear trend. BTMed has prevented approximately 2911 premature deaths among our participants through 2021 and added 35,178 years of life, an average of 1.5 years per participant, at a cost of $2757 per year of life saved.

**Conclusions:**

Secondary prevention in occupational high‐risk groups is very effective. Continued surveillance beyond retirement age is important to reduce mortality.

## 1 Background

The Building Trades National Medical Screening Program is one of five current programs that provide medical exams to workers who have been employed in the US nuclear weapons facilities and are at significantly increased risk for diseases resulting from workplace exposures to radiation and toxic materials. They are funded by the Former Worker Program (FWP), which was authorized by the US Congress in 1993, and medical exams began in 1997 (for additional background see https://www.energy.gov/ehss/former-worker-medical-screening-program-0).

BTMed has been described extensively before. It started with two nuclear weapons facilities and has since grown to cover 35. In 2005, it began to offer follow‐up medical examinations every 3 years; and in 2011, it began to offer early lung cancer detection (ELCD) to a subset at high risk for lung cancer.

The BTMed protocol consists of two steps: (1) a detailed work history interview to establish a participant's exposure risk profile; and (2) an occupational and general preventive medical examination. BTMed participants can choose which program components they wish to participate in. Some individuals choose to participate in the work history interview and delay or forgo a medical exam while the vast majority complete an initial medical exam and follow‐up medical exams approximately every 3 years.

An earlier evaluation of BTMed found that medical exam participants had clinical findings that were significantly improved between the initial medical exam and follow‐up medical exams, including measures of total serum cholesterol; non‐high‐density lipoprotein (non‐HDL) cholesterol; hemoglobin A1c; hypertension; current cigarette smoking; and 10‐year cardiovascular disease (CVD) risk scores, suggesting a substantial impact of secondary prevention [1]. Additionally, mortality follow‐up through December 2021 found reduced all‐cause mortality among BTMed medical exam participants (SMR = 1.15, 95% CI = 1.12–1.18) compared to those only completing the work history interview (SMR = 1.46, 95% CI = 1.38–1.54), with both compared to the US general population [2]. While the ratio of all‐cause SMRs for medical exam participants to work‐history‐only participants (1.12/1.46 = 0.79) is a useful approximation of the magnitude of reduced mortality risk due to medical exam participation, this measure may be inaccurate if the distribution of person‐years by age, gender, or race/ethnicity varies substantially between the two groups. The current study explored differences in mortality risk among medical exam participants and work‐history‐only participants in more detail using Cox regression and Poisson regression models. The following hypotheses were tested:

Hypothesis 1 BTMed medical exam participants experience lower mortality risks for all‐causes and selected causes compared to work‐history‐only participants owing to early disease detection, treatment, and management.

Hypothesis 2 The mortality benefits of BTMed medical exam participation are greater among those continuing in the program, completing follow‐up medical exams beyond the initial medical exam.

Hypothesis 3 Among BTMed medical exam participants, those undergoing more medical exams beyond the initial exam experience greater mortality risk reduction compared to those completing only an initial medical exam.

Hypothesis 4 BTMed participants also participating in the ELCD program experience greater mortality risk reduction compared to those undergoing only medical exams.

Hypothesis 5 The impacts hypothesized above have resulted in a significant number of premature deaths prevented, adding a substantial number of years of life to the BTMed population, resulting in a cost‐effective public health intervention.

## 2 Methods

### 2.1 BTMed Medical Surveillance Program

The BTMed medical surveillance program has been described in prior publications [1, 3, 4, 5]. Briefly, the protocol includes: (1) a detailed work history interview and (2) an occupational and general preventive medical examination. The medical exam consists of a medical history and symptom questionnaire; a smoking history; a physical medical examination; a posterior‐anterior (P‐A) chest radiograph classified by a B‐reader according to International Labour Office (ILO) Classification of Radiographs of Pneumoconiosis [6, 7]; audiometry; a panel of blood tests to measure general clinical parameters as well as specific tests chosen to assess end‐organ damage from identified toxicants; a blood beryllium‐lymphocyte proliferation test (Be LPT, a blood test for beryllium sensitization and potentially chronic beryllium disease [CBD]); a fecal occult blood test (FOBT) for detection of colorectal cancer (CRC); and spirometry meeting American Thoracic Society standards [8, 9]. Workers are eligible for repeat medical exams every 3 years.

BTMed contracts with approximately 250 clinics across the USA that administer the required procedures in accordance with the BTMed protocol. Each medical exam is initiated through a preauthorization issued by BTMed which details the procedures that the clinics are to deliver. After each medical exam, the results are evaluated by a group of specially trained nurses supported by our medical directors to determine the quality and interpretation of each procedure and recommended follow‐up actions. Follow‐up recommendations are noted in the results letters sent to participants, and information materials relevant to follow‐up are included. For significant or urgent findings our nurses also contact participants by phone.

The protocol has undergone several updates since startup in 1997. Total serum non‐HDL cholesterol was added in 2007 and hemoglobin A1c (HbA1c) for diabetes screening was added in 2009. In 2011 BTMed established an ELCD program using low‐dose CT (LDCT) with eligibility based on age, smoking, occupational exposures, and prevalent respiratory diseases [4, 10]. ELCD program participants with continued eligibility are provided with repeat annual LDCT screenings.

### 2.2 Mortality Follow‐Up, Clinical Measures, and Comorbidities

The BTMed cohort has been followed for mortality outcomes through December 31, 2021. Ascertainment of cohort vital status and underlying International Classification of Disease (ICD) causes of death using the US National Death Index (NDI) following procedures previously described [2]. Vital status and underlying cause of death were provided by the NDI Plus system [11]. Workers not identified as deceased by the NDI were assumed alive as of December 31, 2021, based on established completeness of the NDI [12, 13].

Demographic data, clinical measures, and prevalent comorbidities for the current study were taken from the BTMed work history, respiratory symptom questionnaire, initial medical exam, and medical history. Spirometry results were interpreted using prediction equations for the US population [14] and chronic obstructive pulmonary disease (COPD) was defined as a FEV_1_/FVC ratio below the lower limit of normal (LLN) [15, 16]. A lung parenchymal abnormality was defined as the presence on chest radiograph of a small opacity (< 1 cm) of any size or shape with a profusion score ≥ 1/0 using ILO B‐readings [17].

Cigarette smoking history (smoking status and pack‐years), body mass index (BMI) and worker‐reported physician‐diagnoses of hypertension, CVD, diabetes, stroke, or personal history of cancer were taken from the physical examination and medical history.

A history of having smoked was based on a ‘Yes’ response to the question—‘Have you ever smoked cigarettes? (NO means less than 20 packs of cigarettes or 12 oz. of tobacco in a lifetime or less than 1 cigarette a day for 1 year)’. Dyspnea was defined as “Yes” to “Do you walk slower than people your age because of breathlessness?” or a positive answer to additional questions showing more severe shortness of breath. Alcohol consumption was taken from the medical history questionnaire, with heavy alcohol consumption defined as > 2 drinks per day [18].

The work history includes questions concerning a prior diagnosis of asbestosis, silicosis, cancer, or hearing loss, and the prevalence of these diagnoses was compared by medical exam participation status. Additionally, qualitative exposure indices for commonly reported exposures in this population, such as asbestos, silica, cement/concrete dust, organic solvents, and welding/cutting, were developed using the work history, which allowed comparisons between medical exam participants and those only completing the work history. These exposure indices were based on worker‐reported frequencies of performing tasks associated with the exposures of interest using procedures previously described [5, 19]. We also developed an overall measure of exposures to vapors, gases, dusts, and fumes (VGDF Index) to assess combined exposures to the agents listed above.

### 2.3 Assessment of Potential Selection Bias

Self‐selection into the medical exam arm of this study may have been influenced by demographic factors, health status, occupational exposures, or the presence of comorbidities, potentially introducing selection bias. Such bias could either exaggerate or underestimate observed risk estimates. While the work history interview provided useful data for assessing and adjusting for selection bias related to demographics, work history, prevalent work‐related diseases, and self‐reported exposures, it did not include other comorbidity data such as history of hypertension, diabetes, chronic bronchitis or emphysema (COPD), or stroke. Some individuals who completed an initial medical exam delayed the exam, sometimes for a year or more, after the work history interview. To provide additional insight into possible selection bias by medical exam participation status, we compared the demographic data, clinical outcomes, and comorbidities of these ‘late entry’ medical exam participants to those completing the initial medical exam more proximal to their work history interview. We also evaluated the all‐cause mortality experience of individuals completing initial medical exam more than 12 months following the work history interview.

### 2.4 Statistical Analyses

#### 2.4.1 Comparison of Medical Exam Participants to Work‐History‐Only Participants

We compared demographics, work history, reported diagnoses of asbestosis, silicosis or cancer, and qualitative exposure indices for BTMed medical exam participants to those only completing the work history interview. We also compared demographic characteristics, clinical findings, and comorbidities for medical exam participants completing an initial medical exam within 1 year of the work history interview and those completing an initial medical exam more than 12 months after the work history interview. Categorical variables were tabulated by frequency and prevalence (percent); means and standard deviations (STD) were calculated for continuous variables. Median and interquartile ranges (IQR) were calculated for continuous variables with a significantly skewed distribution. Continuous variables were compared by analysis of variance (ANOVA). Wilcoxon rank‐sum tests were employed for comparing continuous variables that departed significantly from a normal distribution. Categorical variables were compared using a Chi‐square test of general association.

The analyses of standardized mortality ratios (SMR) in the current study followed procedures described in our prior publication [2]. Briefly, the Life Table Analysis System (LTAS) developed by the National Institute for Occupational Safety and Health (NIOSH) and implemented as an R‐Statistical Package [20] was utilized to compute cause‐specific SMRs and statistical confidence intervals to compare the mortality experience of the cohort to that of the US national population, adjusting for age (5‐year age categories), race (White and non‐White), gender, and calendar year (5‐year calendar time periods). Person‐years accumulation began on the date of the work history interview for work‐history‐only participants and at initial medical exam for BTMed medical exam participants and was accumulated until death or the study cut‐off date of December 31, 2021. All analyses were based on the death certificate underlying cause of death.

We summarized SMRs for all‐causes and specific causes of death for medical exam participants and work‐history‐only participants. Major causes of death included all cancers, all respiratory diseases, and all CVDs. Sub‐categories with approximately 100 deaths among work‐history‐only participants were summarized and included lung cancers, CRCs, COPD, and ischemic heart disease.

In addition to the comparisons of SMRs among medical exam participants and work‐history‐only participants, competing‐causes Cox proportional hazard regression models were developed for the causes listed above, which allowed control for age, gender, race/ethnicity, history of work in construction trades, and a prior diagnosis of asbestosis, silicosis, or cancer. Attained age was used as the time scale in the Cox models. Kaplan‐Meier product‐limited survival curves comparing medical exam participants to work‐history‐only participants were generated for visualization of survival differences.

All‐cause mortality risk by extent of BTMed medical exam participation was investigated using Cox models with a time‐varying measure of the number of completed BTMed medical exams. These models adjusted for age, gender, race/ethnicity, history of work in construction trades and a prior diagnosis of asbestosis, silicosis, or cancer.

We used Cox proportional hazards models to assess how follow‐up medical exam participation and ELCD program participation affected all‐cause mortality among individuals completing an initial BTMed medical exam. These models incorporated a time‐varying measure of the number of medical exams completed and ELCD program participation and adjusted for age, gender, race/ethnicity, smoking status, history of work in construction trades, and comorbidities at initial medical exam including history of hypertension, diabetes, chronic bronchitis or emphysema (COPD), stroke, and cancer.

#### 2.4.2 Assessing Delay of Exam Impact

While information concerning history of hypertension, diabetes, chronic bronchitis or emphysema (COPD), and stroke was unavailable for those completing only the work history interview, we analyzed the all‐cause mortality experience for those completing an initial medical exam more than 12 months since their work history interview to gain additional insight about the direction of potential bias caused by self‐selection into the medical exam program. We used the all‐cause Cox model for these analyses with a categorical covariate for work‐history‐only, initial medical exam within one year of completing the work history, or initial medical exam more than 12 months since the work history. This model adjusted for age, gender, race/ethnicity, history of work in construction trades, and a prior diagnosis of asbestosis, silicosis, or cancer.

#### 2.4.3 Definition of Impact Measurements

We defined premature mortality as the difference in deaths observed among medical exam participants and predicted deaths assuming the mortality experience of medical exam participants would be approximately the same as for the work‐history‐only participants, in the absence of BTMed medical exam participation. Predicted deaths were estimated using the Poisson regression model for all‐cause mortality. The Poisson regression model was used to score medical exam participants using model coefficients for work‐history‐only participants to estimate expected deaths among medical exam participants under the counterfactual assumption that they died at the same model adjusted rates as work‐history‐only participants. Premature deaths averted were calculated as the difference between deaths observed among medical exam participants and expected deaths calculated under the counterfactual assumption.

Years of life added by medical exam participation were estimated using restricted mean survival time (RMST) regression methods [21, 22]. RMST regression estimates survival until a specified time while accounting for censoring and adjusting for risk factors and confounders. RMST is calculated as the area under the adjusted survival curves. We used the SAS Root Mean Survival Time Regression (RMSTREG) procedure to estimate mean survival times for medical exam participants and work‐history‐only participants while controlling for age, gender, race/ethnicity, history of work in construction trades, and a prior diagnosis of asbestosis, silicosis, or cancer. The survival time window was defined as the largest event time (23.8 years) for the cohort to capture all cohort data. The difference between the model RMST values for medical exam participants and work‐history‐only participants provided an estimate of the mean years of life gained due to medical exam participation. Total years of life added to the cohort due to medical exam participation was estimated by multiplying the number of medical exam participants by the mean years of life gained due to medical exam participation. Cost per year of life saved was calculated by summing total program costs from 1997 to 2021 (24 years) and dividing it by the estimated years of life saved.

#### 2.4.4 Sensitivity Analyses

Sensitivity analyses by analytical method were performed for each mortality outcome analyzed. Comparable Poisson regression models of rate‐ratios were developed for each Cox time to event model. We also conducted sensitivity analyses whereby the start of follow‐up was delayed by 6 months to address potential selection bias associated with possible terminal illness present at cohort entry. We used the Cox all‐cause mortality model for these analyses.

Statistical analyses used SAS/STAT 9.4 [23] and the LTASR R‐Statistical Package [20]. *p*‐values < 0.05 were considered statistically significant.

### 2.5 Human Subjects Protection

All study procedures and materials were reviewed and approved by the Central Department of Energy Institutional Review Board (CDOE IRB). All participants provided informed consent. The use of data for the current analyses was approved by the CDOE IRB (DOE001263 approved August 18, 2025).

## 3 Results

**Figure 1:**
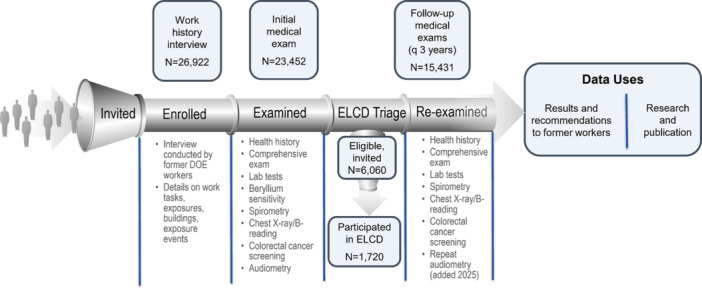
Participants and data sources for BTMed program impact analyses.

A schematic summary of participants' data sources for this study is provided in Figure 1. A total of 26,922 were included in the study with 23,452 BTMed medical exam participants and 3470 work‐history‐only participants. The BTMed medical exam participants completed 15,431 follow‐up medical exams through December 31, 2021 and 1720 workers also participated in the ELCD program.

Table 1 provides a summary of demographic characteristics of the study cohort by BTMed medical exam participation status at cohort entry. BTMed medical exam participants were slightly younger than those completing only a work history, although the difference in mean values was small (< 1 year). There were no significant differences by gender or race/ethnicity. Medical exam participants were significantly more likely to have been employed in construction and had significantly more years of work in construction trades and on DOE sites. There were no significant differences in the prevalence of reported prior diagnoses of asbestosis, silicosis, cancer, or hearing loss by medical exam participation status. The median exposure indices for asbestos, silica, cement/concrete dust, organic solvents, welding/cutting and the summary VGDF index were significantly higher for medical exam participants compared to work‐history‐only participants.

**Table 1:** Demographic characteristics by medical exam participation status.

Measure	Medical exam participants (*N* = 23,452)	Work‐history‐only participants (*N* = 3470)	*p* value^a^
Age, mean (STD)^b^	60.1 (12.2)	61.0 (13.6)	0.0011
Gender, *N* (%)			
Male	21,747 (92.6)	3208 (92.4)	0.6818
Female	1725 (7.4)	262 (7.6)	
Race/Ethnicity			
White	20,368 (86.9)	3021 (87.1)	0.7214
Non‐White	3084 (13.1)	449 (12.9)	
Construction trade work, *N* (%)	19,991 (85.2)	2756 (79.4)	< 0.0001
Years of construction work (mean, STD)	21.5 (15.1)	19.3 (15.4)	< 0.0001
Years of DOE site work (mean, STD)	9.2 (10.2)	7.0 (8.9)	< 0.0001
Worker reported prior diagnoses, *N* (%)^c^			
Asbestosis	901 (4.0)	131 (4.0)	0.9976
Silicosis	71 (0.3)	11 (0.3)	0.8381
Cancer	3185 (14.1)	481 (14.6)	0.3716
Hearing loss	6437 (32.3)	991 (33.4)	0.2798
Occupational exposure indices, median (IQR)^d^			
Asbestos	6.1 (0.5–28.7)	3.0 (0–18.4)	< 0.0001
Silica	10.9 (2.6–32.2)	7.5 (0.9–27.5)	< 0.0001
Cement/concrete dust	10.8 (1.8–34.5)	6.0 (0.3–33.0)	< 0.0001
Organic solvents	8.1 (2.2–19.5)	6.3 (1.0–16.5)	< 0.0001
Welding/cutting	12.6 (2.5–39.7)	7.5 (0.9–33.5)	< 0.0001
VGDF^e^	74.4 (24.1–143.0)	56.8 (12.9–125.0)	< 0.0001

^a^ *p*‐Value comparing medical exam participants to work‐history‐only participants.

^b^ STD = standard deviation.

^c^ Data was not available for 977 individuals. Percentages are based on those with data.

^d^ IQR = interquartile range.

^e^ Index for collective exposures to vapors, gases, dusts, and fumes (VGDF). The VGDF index is the sum of indices for asbestos, silica, organic solvent, cement/concrete dust, and welding/cutting for each worker.

Characteristics of medical exam participants stratified by time between the work history interview and the initial medical exam are compared in Table 2. The median time from work history interview to initial medical exam was one month for those completing the initial medical exam within 12 months of the work history and 24 months for those choosing to enter the medical exam program later. Individuals delaying the initial medical exam were slightly younger, and the percentage of males was slightly higher, although the absolute magnitude of these differences was small. Those delaying the initial medical exam had a higher prevalence of individuals currently smoking and employed in construction trades. However, the prevalence of hypertension, CVD, stroke, and dyspnea were higher among those completing the initial medical exam soon following the work history interview. The prevalence of chest X‐ray (CXR) parenchymal changes and COPD by spirometry was not significantly different between the two groups. Those completing the initial medical exam early had significantly more years of construction trade work and years of work on DOE sites.

**Table 2:** Characteristics of medical exam participants by time between the work history interview and the initial medical exam.

Characteristic^a^	All medical exam participants (*n* = 23,452)	Participants entering < 12 months after work history interview (*n* = 21,694)	Participants entering ≥ 12 months after work history interview (*n* = 1758)	*p* value^b^
Months from work history interview to initial medical exam (median, IQR)	1.0 (1.0–3.0)	1.0 (1.0–2.0)	24.0 (15.0–42.0)	
Age (years, mean, STD)	60.1 (12.2)	60.2 (12.3)	59.6 (11.9)	0.0464
Sex				0.0341
Male	21,727 (92.6)	20,076 (92.5)	1651 (93.9)	
Female	1725 (7.4)	1618 (7.5)	107 (6.1)	
Race/Ethnicity				0.9521
White	20,368 (86.9)	18,842 (86.9)	1526 (86.8)	
Non‐white	3084 (13.1)	2852 (13.2)	232 (13.2)	
Smoking status				< 0.0001
Never	8310 (35.3)	7735 (35.6)	575 (32.7)	
Former	10,542 (45.0)	9777 (45.1)	765 (43.5)	
Current	4381 (18.7)	4003 (18.5)	378 (21.5)	
Unknown	219 (0.9)	179 (0.8)	40 (2.3)	
Smoking pack‐years (mean, STD)	20.7 (25.7)	20.6 (25.6)	22.0 (26.9)	0.0312
BMI (mean, STD)	29.8 (5.6)	29.8 (5.6)	29.8 (5.5)	0.9555
Hypertension history	12,183 (52.0)	11,329 (52.2)	854 (48.9)	0.0033
Diabetes history	4093 (17.5)	3813 (17.6)	280 (15.9)	0.0798
Cardiovascular disease history	11,851 (50.5)	11,017 (50.8)	835 (47.5)	0.0081
Stroke history	878 (3.7)	827 (3.8)	51 (2.9)	0.0529
Personal cancer history	5302 (22.6)	4966 (22.9)	336 (19.1)	0.0003
Bronchitis/emphysema history	2830 (12.1)	2634 (12.1)	196 (11.2)	0.2192
Heavy alcohol consumption (> 2 drinks/day)	2816 (12.0)	2596 (12.0)	220 (12.5)	0.4968
Dyspnea history	8099 (34.5)	7571 (34.9)	528 (30.0)	< 0.0001
COPD by spirometry	3343 (14.9)	2997 (14.5)	246 (15.2)	0.4725
CXR parenchymal profusion ≥ 1/0	900 (4.0)	830 (4.0)	70 (4.3)	0.5467
Construction trade work	19,991 (85.2)	18,432 (85.0)	1559 (88.7)	< 0.0001
Years of construction work (mean, STD)	21.6 (15.1)	21.7 (15.2)	19.4 (14.0)	< 0.0001
Years of DOE site work (mean, STD)	9.2 (10.2)	9.1 (10.2)	8.5 (9.2)	< 0.0001
**Deaths from all causes**	**7223**	**6650**	**573**	

^a^ Data are N (%) or mean (standard deviation, STD), or median (interquartile range, IQR) unless otherwise specified. Some individuals did not choose to participate in the spirometry, chest X‐ray, or the audiometry portion of the medical exam and the numbers and percentages are based on individuals with data.

^b^ *p*‐Values compare medical exam participants who did not delay the medical exam and those who delayed the medical exam by ≥ 12 months.

**Figure 2:**
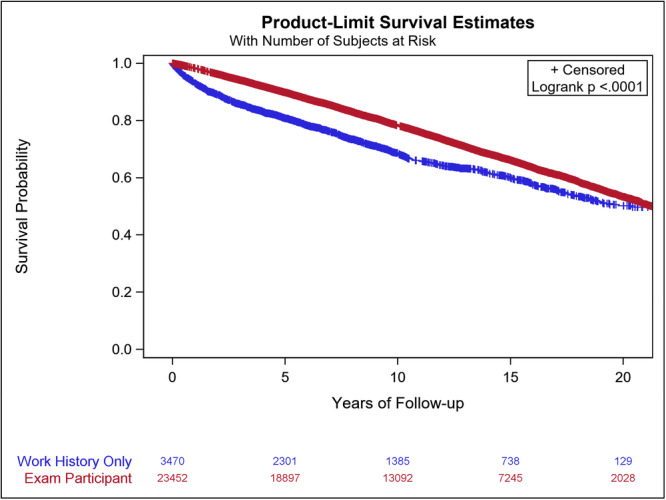
Kaplan‐Meier survival curves for medical exam participants and work‐history‐only participants.

Figure 2 provides the Kaplan‐Meier survival plots for medical exam participants and work‐history‐only participants, demonstrating substantial differences in survival (*p* < 0.0001). The SMR comparisons by cause of death among medical exam participants and work‐history‐only participants are summarized in Table 3. Results of the Cox and Poisson models are also presented in this table. The SMRs for all causes analyzed were lower among medical exam participants compared to work‐history‐only participants.

**Table 3:** Mortality by BTMed medical exam participation status.

Cause of death	BTMed medical exam status	Observed deaths	SMR (95% CI)^a^	Cox model hazard ratio (95% CI)^b^	Poisson regression model rate ratio (95% CI)^b^
All causes	Work‐history‐only	1144	1.46 (1.38–1.54)	1.00 (Ref)	1.00 (Ref)
	Medical exam participant	7222	1.15 (1.12–1.18)	0.72 (0.68–0.77)	0.71 (0.67–0.76)
All cancers	Work‐history‐only	318	1.70 (1.52–1.90)	1.00 (Ref)	1.00 (Ref)
	Medical exam participant	1985	1.31 (1.25–1.37)	0.68 (0.61–0.77)	0.69 (0.61–0.78)
Lung cancer	Work‐history‐only	98	1.99 (1.61–2.42)	1.00 (Ref)	1.00 (Ref)
	Medical exam participant	607	1.49 (1.37–1.61)	0.64 (0.52–0.80)	0.67 (0.54–0.83)
Colorectal cancer	Work‐history‐only	33	2.04 (1.40–2.86)	1.00 (Ref)	1.00 (Ref)
	Medical exam participant	137	1.05 (0.88–1.24)	0.47 (0.32–0.69)	0.47 (0.32–0.69)
All respiratory diseases	Work‐history‐only	152	1.95 (1.65–2.29)	1.00 (Ref)	1.00 (Ref)
	Medical exam participant	989	1.54 (1.45–1.64)	0.73 (0.62–0.87)	0.74 (0.62–0.88)
COPD	Work‐history‐only	100	2.25 (1.83–2.74)	1.00 (Ref)	1.00 (Ref)
	Medical exam participant	568	1.54 (1.41–1.67)	0.63 (0.51–0.78)	0.64 (0.52–0.79)
All cardiovascular diseases	Work‐history‐only	342	1.31 (1.18–1.46)	1.00 (Ref)	1.00 (Ref)
	Medical exam participant	2078	0.99 (0.95‐1.03)	0.70 (0.63–0.79)	0.68 (0.61–0.77)
Ischemic heart disease	Work‐history‐only	174	1.30 (1.11–1.50)	1.00 (Ref)	1.00 (Ref)
	Medical exam participant	1057	0.97 (0.71–1.25)	0.69 (0.59–0.81)	0.68 (0.58–0.80)
All other causes	Work‐history‐only	332	1.28 (1.14–1.43)	1.00 (Ref)	1.00 (Ref)
	Medical exam participant	2171	1.05 (1.00–1.09)	0.77 (0.69–0.86)	0.73 (0.65–0.83)

^a^ SMRs based on US death rates by gender, race/ethnicity and 5‐year categories of age and calendar time‐period.

^b^ All models adjusted for age, gender, race/ethnicity, employment in construction trades and a prior diagnosis of asbestosis, silicosis, or cancer. Attained age used as the Cox model time scale.

The all‐cause mortality hazard ratio (HR) for medical exam participation compared to those work‐history‐only participants was 0.72 (95% CI = 0.68–0.77). The HRs for medical exam participants were significantly reduced across all causes of death analyzed ranging from 0.47 for CRC to 0.77 for all‐other causes. The Poisson regression models produced mortality rate ratios (RR) comparable to the Cox model results for all causes analyzed. The sensitivity analyses delaying cohort follow‐up by 6 months following cohort entry produced a comparable HR for all‐causes in the Cox model (HR = 0.79, 95% CI = 0.74–0.85).

More in‐depth analyses of all‐cause mortality are provided in Table 4. Compared to those completing only the work history interview, the mortality risk decreased with the number of medical exams completed beyond initial exam with a significant trend (*p* < 0.0001). The HR among those completing only an initial medical exam was 0.75 (95% CI = 0.70–0.81) whereas the HR was reduced to 0.57 (95% CI = 0.51–0.73) among those completing three or more BTMed medical exams.

**Table 4:** All‐cause mortality by BTMed medical exam participation metrics.

All‐cause mortality comparisons	BTMed medical exam participation measure	Observed deaths	Cox model hazard ratio (95% CI)	Poisson regression model rate ratio (95% CI)
Number of BTMed medical exams vs. work‐history‐only	Work‐history‐only	1144	1.00 (Ref)^a^	1.00 (Ref)^a^
	One BTMed medical exam	4758	0.75 (0.70–0.81)	0.74 (0.70–0.79)
	Two BTMed medical exams	1850	0.71 (0.66–0.77)	0.70 (0.65–0.75)
	Three or more BTMed medical exams	615	0.57 (0.52–0.63)	0.56 (0.50–0.61)
Number of follow‐up medical exams among medical exam participants	Initial medical exam only	4758	1.00 (Ref)^b^	1.00 (Ref)^b^
	Two BTMed medical exams	1850	1.00 (0.94–1.05)	1.00 (0.94–1.05)
	Three or more BTMed medical exams	615	0.82 (0.75–0.89)	0.83 (0.76–0.90)
BTMed medical exam participants and ELCD participants	Medical exam participant only	7003	1.00 (Ref)^b^	1.00 (Ref)^b^
	Medical exam and ELCD participant	219	0.65 (0.57–0.74)	0.64 (0.56–0.74)

^a^ Models adjusted for age, gender, race/ethnicity, employment in construction trades and a prior diagnosis of asbestosis, silicosis, or cancer. Attained age used as the Cox model time scale.

^b^ Model adjusted for age, gender, race/ethnicity, smoking status, employment in construction trades, and history of hypertension, diabetes, chronic bronchitis or emphysema (COPD), stroke, and cancer. Attained age used as the Cox model time scale.

Table 4 also provides analyses confined to those completing one or more BTMed medical exams. These analyses allowed for control of additional risk factors and comorbidities. The Cox model found a significant trend in reduced all‐cause mortality with increasing BTMed medical exam participation (*p* < 0.0001). Those completing three or more BTMed medical exams were at significantly reduced mortality compared to those completing only an initial medical exam (HR = 0.82, 95% CI = 0.75–0.89).

Participation in the ELCD program further reduced the all‐cause mortality risk (Table 4). Among BTMed medical exam participants, those also participating in the ELCD program were at significantly reduced all‐cause mortality compared to BTMed medical exam participants alone (HR = 0.65, 95% CI = 0.57–0.74). Poisson regression model results were entirely consistent with the Cox model findings. Detailed output for all Cox models for causes of death in Tables 3 and 4 are provided in the Supplemental Materials.

The Cox model examining all‐cause mortality in relation to medical exam participation and time since completing the work history interview found a comparable magnitude of reduced mortality for those completing the initial medical exam within 1 year of the work history interview (HR = 0.72, 95% CI = 0.68–0.77) and those delaying the initial medical exam for more than 12 months (HR = 0.75, 95% CI = 0.68–0.83). Detailed results for this model are provided in the Supporting Information Materials S1.

There were significant differences in the Kaplan‐Meier functions comparing medical exam participants and work‐history‐only participants (Figure 2 described above). Using the Poisson model for all‐causes and our counterfactual methods, we estimated that the program has resulted in 2911 deaths prevented through 2021. The RMST model estimated the adjusted mean survival time for medical exam participants after cohort entry to be 16.7 (95% CI = 16.6–16.9) years compared to 15.2 (95% CI = 14.9–15.5) years among those completing only the work history interview. The difference between these values, 1.5 years, is the estimated adjusted mean years of life added per medical exam participant. Based on these differences in survival we estimate that the program has resulted in 35,178 years of life being added to those who participated in the medical examinations through 2021.

Total cost of the program from start through 2021 was $97,000,000. The estimated cost per year of life saved is $2757 [$97,000,000/35,178 years of life saved]. This is a crude estimate that is not adjusted for inflation since program inception in 1997.

## 4 Discussion

### 4.1 Secondary Prevention and Occupational High‐Risk Management

On May 9, 1977, Dr. John F. Finklea, the Director of the NIOSH, testified before the Subcommittee on Labor, Committee on Human Resources of the US Senate on the activities of his agency: “The primary subject of our testimony today will be a discussion of our efforts to notify workers of health hazards that they may have encountered on their jobs and to assist them in entering the medical care system for early diagnosis and treatment if indicated.” He concluded his testimony by raising a new issue that needed to be considered: “The primary purpose of the Occupational Safety and Health Act of 1970 is to establish standards that will reduce workplace exposures to safety and health hazards so that no worker will suffer diminished health, functional capacity, or life expectancy as a result of his work experience. Until adequate standards are developed, however, we must not forget to provide for the workers suffering as a result of existing workplace conditions” (see https://stacks.cdc.gov/view/cdc/180284).

How to do this was explored over the following decade, and included how to identify and inform workers who have been found to be at high risk of work‐related diseases (either based on exposures and/or health outcomes) as well as the kind of services they should receive [24], which was influenced by developments in occupational medicine at the time. Gradually comprehensive occupational health programs evolved to include aspects of secondary prevention in addition to primary prevention activities (such as elimination or reduction of workplace exposures). Thus, medical surveillance and medical screening became understood to represent complementary secondary preventive components of a comprehensive occupational health program, and this has been applicable to both workplaces and occupational high‐risk programs [25].

The Coal Workers Health Surveillance Program, established in 1969, was an early example of a medical surveillance program, which was developed for a specialized industrial sector (https://www.cdc.gov/niosh/cwhsp/about/index.html). The question of what to do with workers in “general industry” had been raised by Dr. Irving Selikoff and others for inclusion in the Occupational Safety and Health Act in 1970, but they did not prevail, and it continued to be debated for the next decade and a half [24]. A few demonstration projects showed that occupational high‐risk intervention programs could be implemented, but they did not achieve long‐term sustainability [26]. The High‐Risk Occupational Disease Notification and Intervention Act was introduced in the 99th Congress (1985–86) and again in the 100th Congress (1987–88) but was not adopted.

The main obstacle to adopting legislation to address workers at high risk of disease was uncertainty about the magnitude of liability for future occupational diseases, which often would not be manifested until late in life and long after retirement, and who should be responsible for that liability. While Congress found a way to remediate the legacy of toxic hazards from defunct manufacturing and mining sites through the Comprehensive Environmental Response, Compensation, and Liability Act (CERCLA) (1980), it could not find a way to do the same for workers.

Among the high‐risk workplaces identified were the nuclear weapons facilities of the US government. After more than a decade of legislative advocacy and significant litigation, the Former Worker Medical Screening Program was established in 1996, and in 2000 Congress passed the Energy Employees Occupational Illness Program Act for workers and their survivors who had developed work‐related toxic illnesses. Together, the medical screening program and the compensation program formed a comprehensive framework for high‐risk management, coordinated through the US Department of Energy (screening) and US Department of Labor (compensation). The only other federal occupational high‐risk surveillance program adopted since then is the World Trade Center Health Program (https://www.cdc.gov/wtc/about.html) in 2010.

We believe this report provides the most detailed and long‐term assessment of the value of occupational high‐risk management practices.

### 4.2 BTMed Impact

The impact of a medical surveillance program is a function of the predictive value of the procedures administered in relation to the risk profile of the population served, and the ability of the program to achieve adherence with testing schedules and test follow‐up recommendations [27, 28, 29]. Our previous evaluation found significantly improved clinical findings for measures of total serum cholesterol; non‐high density lipoprotein (non‐HDL) cholesterol; hemoglobin A1c hypertension; current cigarette smoking; and 10‐year CVD risk scores between initial medical exam and follow‐up medical exam [1]. All BTMed participants were offered CRC screening at the start of the program due to their significant asbestos exposure, which at that time had been linked—though not definitively—to increased risk [30]. A recent review and meta‐analysis found strong evidence of increased risk of CRC among individuals occupationally exposed to asbestos [31], supporting BTMed's early decision on CRC screening. A recent evaluation found that BTMed achieved a high rate of CRC screening participation and significantly reduced CRC mortality [32]. Our data support the conclusion that CRC mortality in our population is decreased as a result of BTMed medical exam participation. Likewise, BTMed lung cancer screening has been found effective in detecting lung cancers at an early stage and reducing lung cancer mortality risk [10, 33]. In both cases, adherence to follow‐up recommendations has been very good. This, combined with a consistent nearly‐100% satisfaction response from our participants, are indicators that the program is administered well and accepted by our participants.

The current study provides further evidence of decreased all‐cause mortality and mortality from major categories of death among BTMed medical exam participants. There was evidence to support each of the stated study hypotheses, including greater benefit of continued participation through follow‐up medical exams and impacts of program components such as lung cancer screening. The BTMed program has prevented premature deaths and added years of life to the population of participants. We calculated crudely (without adjusting for inflation) the mean cost per year of life saved to be $2757. In a 1995 study of 587 life‐saving health interventions that saved more than they cost, Tengs et al. found that the median intervention cost was $44,000 per year of life saved for all types of interventions, $19,000 for all medical interventions, and $23,000 for secondary prevention interventions. The BTMed costs per year of life compared favorably to the most cost‐effective interventions they studied, such as prenatal care ($2100) and influenza vaccination for people over age 5 ($1300) [34].

### 4.3 Value of Health Screening

There has been a long and simmering debate about the value of periodic health screenings in general and in occupational populations, and our findings show much greater impact than reported elsewhere. There are few other published studies with which to compare. A 2014 meta‐analysis of six clinical trials involving general health checkups found a small but significant impact of continued surveillance, particularly in high‐risk groups, based on surrogate measures of outcomes (e.g., blood pressure, BMI, etc.) [35]. While a 2019 Cochrane review of 17 clinical trials comparing people receiving medical check‐ups to controls did not find a significant impact on mortality outcomes, this review was limited in that it included several outdated studies (some from the 1960s); included studies focused on differing check‐up procedures; excluded studies with participants over age 65; did not include projects aimed at high‐risk groups; and did not focus primarily on groups defined by occupation. Five of the trials were focused on screening for cancer but used what are now considered to be obsolete procedures such as CXRs for lung cancer screening, early‐generation stool blood tests, rigid sigmoidoscopy, etc., as interventions. One‐third of the reviewed trials included a physical medical examination by a physician while two‐thirds did not [36].

Most published studies assessing impacts of workplace health surveillance and health promotion programs have focused on measures of physical and mental well‐being using short‐term outcome measures such as absenteeism, job satisfaction, alcohol and tobacco use, dietary and exercise habits, obesity, and others [37, 38]. Similar to the earlier BTMed evaluation, some studies have shown positive impacts of workplace interventions to identify undiagnosed hypertension, diabetes, and hyperlipidemia [39, 40, 41]. Few studies have examined the impact of comprehensive occupational health surveillance exams on long‐term mortality. Significantly reduced mortality was observed among chemical plant shift workers participating in an occupational health promotion program consisting of occupational medical exams and health seminars [42, 43]. Over an average of 7.66 years of mortality follow‐up, the all‐cause mortality relative‐risk among participants was 0.83 (95% CI = 0.69–0.99) after adjustment for confounders, including smoking, alcohol consumption, and comorbidities.

**Figure 3:**
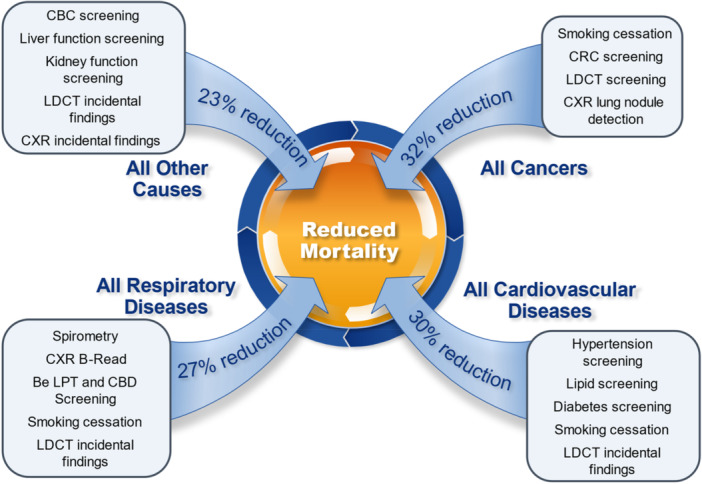
Premature mortality reduction by cause of death and medical exam elements likely contributing to reductions.

The BTMed program focuses on occupational medical examinations and surveillance while also providing participants with other medical tests and more general medical surveillance. While occupational respiratory diseases and cancers have been an important emphasis of BTMed, the surveillance exams address multiple organ systems and outcomes, whose collective impact was assessed in this study. Medical exam components are not independent, and some components may affect mortality for more than one cause. For medical example, detection and control of diabetes and hypertension may reduce CVD mortality as well as mortality due to kidney diseases. Likewise, lung cancer screening with LDCT often identifies other incidental findings such as non‐malignant lung or pleural disease, coronary artery calcification, thyroid nodules, adrenal nodules, thymus nodules or masses, aortic abnormalities including aneurysm, liver or kidney nodules, hiatal hernia, or lymphadenopathy, some of which are amenable to management or treatment [44]. Figure 3 provides a conceptional summary of mortality reductions by major causes observed in the BTMed cohort and medical exam components likely contributing to these reductions; without quantifying the attribution of each component.

### 4.4 Strengths and Limitations

This study has several strengths and limitations. An important strength is the availability of a large, older, high‐risk population participating in an occupational health surveillance program with long‐term mortality follow‐up. Mortality follow‐up of the subpopulation of work‐history‐only participants provided a unique opportunity for mortality comparisons while controlling for demographic characteristics, work history variables, and prior diagnoses of selected comorbidities.

A key limitation of this study is its observational design and the potential for selection bias related to medical exam participation. Although we were able to adjust for demographic characteristics, work history, and prior diagnoses of selected comorbidities between the study arms, data on a broader list of clinical measures and comorbidities were not available for individuals who completed only the work history component. However, the work‐history‐only group was less likely to be employed in construction trades, had comparable histories of asbestosis, silicosis, and cancer and had lower exposure indices for asbestos, silica, organic solvents, and welding/cutting activities—factors that would reduce the likelihood of our study overestimating any protective effect. Moreover, comparisons between participants who completed the initial medical exam within one year of the work history interview and those who did so after more than a year revealed no substantial differences in the HR for all‐cause mortality. Additionally, participants completing more than an initial medical exam demonstrated decreased all‐cause mortality compared to initial medical exam only participants. Taken as a whole, the available data do not suggest systematic differences in underlying risk factors or comorbidities between medical exam participants and work‐history‐only participants that would meaningfully alter study results.

## 5 Conclusions

This study found substantial mortality reductions in a high‐risk population of older individuals who participated in a comprehensive occupational medicine surveillance program. Long‐term follow‐up is important in assessing the impact of such programs, but few published studies extend beyond retirement age; therefore, there are few studies to use for comparison. This study demonstrates the importance of continued surveillance of occupational diseases in high‐risk populations beyond retirement age to reduce mortality.

## Author Contributions

Knut Ringen and John Dement co‐generated study hypotheses and study analytical design, and John Dement conducted the statistical analyses and prepared of the tables and Figure 2. Marianne Cloeren prepared Figures 1 and 3. John Dement and Knut Ringen led the data interpretation, wrote the first draft of the manuscript, and contributed to its editing and revision. Marianne Cloeren, Sammy Almashat, William Grier, Stella Hines, Laura Welch, and Kim Cranford contributed to acquisition of the medical data, provided clinical data interpretation, and contributed to manuscript editing and revision. Scott Haas, Patricia Quinn and Anna Chen played a key role in medical data collection, management, and quality control and participated in manuscript editing and revision. Miles Fisher contributed to subject recruitment. All authors contributed to the planning, execution, and reporting of the work and approved the final manuscript. Knut Ringen, as both the guarantor and corresponding author, ensured the accuracy and integrity of the research.

## Disclosure

The opinions expressed in this publication are those of the authors and do not express the views of the U.S. Department of Energy.

## Ethics Statement

All study procedures and materials were reviewed and approved by the Central Department of Energy Institutional Review Board (CDOE IRB). The use of data for the current analyses was approved by the CDOE IRB (DOE001263, approved August 18, 2025).

## Consent

All participants provided informed consent.

## Conflicts of Interest

The authors declare no conflicts of interest.

## Supporting information

AJIM‐9937707 R1 Supplemental Materials 12 22 2025.

## Data Availability

The authors have nothing to report.
