# Supplementary material for: Impact of Secondary Prevention on Mortality in the Building Trades National Medical Screening Program: Effectiveness of Occupational High‐Risk Management
Source: Am J Ind Med. 2026 Jan 6;69(4):241–53. doi: 10.1002/ajim.70052 (PMC12981205; doi:10.1002/ajim.70052)
Supplement: Supplementary file 1 — AJIM‐9937707 R1 Supplemental Materials 12 22 2025. [file AJIM-69-241-s001.pdf]

SUPPLEMENTAL MATERIALS

Table of Contents

Cox Proportional Hazard Model Results by Mortality Outcome (Manuscript Table 3) 2
All-Cause Mortality 2
All Cancer Morality 3
Colorectal Cancer Mortality 4
Lung Cancer Mortality 5
All Respiratory Disease Mortality 6
COPD Mortality 7
All Cardiovascular Disease Mortality 8
Ischemic Heart Disease Mortality 9
All Other Causes Mortality 10
Cox Proportional Hazard Model Results for All-Causes by BTMed Exam Participation Metrics (Manuscript Table 4) 11
Number of BTMed Exams vs Work History Only 11
Number of Follow-up Exams among Exam Participants 12
BTMed Exam Participants and ELCD Participants 13
Cox Model for All-cause Mortality by Time Since Completing the Work History Interview 14

Cox Proportional Hazard Model Results by Mortality Outcome (Manuscript Table 3)

All-Cause Mortality

Parameter	Value	DF	Parameter Estimate	Standard Error	Chi-Square	Pr > ChiSq	Hazard Ratio	95% Hazard Ratio Confidence Limits	
**Gender**	Female	1	-0.37950	0.06635	32.7103	<.0001	0.684	0.601	0.779
**Gender**	Male (Ref)	0	0	.	.	.	.	.	.
**Race/Ethnicity**	Non-White	1	0.12579	0.03730	11.3730	0.0007	1.134	1.054	1.220
**Race/Ethnicity**	White (Ref)	0	0	.	.	.	.	.	.
**Construction Trade Work**	1	1	0.31410	0.03645	74.2722	<.0001	1.369	1.275	1.470
**Construction Trade Work**	0 (Ref)	0	0	.	.	.	.	.	.
**Exam Participant**	1	1	-0.32696	0.03198	104.5396	<.0001	0.721	0.677	0.768
**Exam Participant**	0 (Ref)	0	0	.	.	.	.	.	.
**Asbestosis, Silicosis or Cancer History**	1	1	0.13938	0.02783	25.0843	<.0001	1.150	1.089	1.214
**Asbestosis, Silicosis or Cancer History**[1]	99[1]	1	0.00156	0.05836	0.0007	0.9787	1.002	0.893	1.123
**Asbestosis, Silicosis or Cancer History**	0 (Ref)	0	0	.	.	.	.	.	.

[1] A value of 99 indicates missing.

All Cancer Morality

Parameter	Value	DF	Parameter Estimate	Standard Error	Chi-Square	Pr > ChiSq	Hazard Ratio	95% Hazard Ratio Confidence Limits	
**Gender**	Female	1	-0.33991	0.12170	7.8014	0.0052	0.712	0.561	0.904
**Gender**	Male (Ref)	0	0	.	.	.	.	.	.
**Race/Ethnicity**	Non-White	1	0.13623	0.06943	3.8501	0.0497	1.146	1.000	1.313
**Race/Ethnicity**	White (Ref)	0	0	.	.	.	.	.	.
**Exam Participant**	1	1	-0.38133	0.06063	39.5537	<.0001	0.683	0.606	0.769
**Exam Participant**	0 (Ref)	0	0	.	.	.	.	.	.
**Construction Trade Work**	1	1	0.44788	0.07248	38.1844	<.0001	1.565	1.358	1.804
**Construction Trade Work**	0 (Ref)	0	0	.	.	.	.	.	.
**Asbestosis, Silicosis or Cancer History**	1	1	0.45993	0.05022	83.8869	<.0001	1.584	1.435	1.748
**Asbestosis, Silicosis or Cancer History**	99[1]	1	-0.00143	0.11431	0.0002	0.9900	0.999	0.798	1.249
**Asbestosis, Silicosis or Cancer History**	0 (Ref)	0	0	.	.	.	.	.	.

[1] A value of 99 indicates missing.

Colorectal Cancer Mortality

Parameter	Value	DF	Parameter Estimate	Standard Error	Chi-Square	Pr > ChiSq	Hazard Ratio	95% Hazard Ratio Confidence Limits	
**Race/Ethnicity**	Non-White	1	-0.00178	0.26626	0.0000	0.9947	0.998	0.592	1.682
**Race/Ethnicity**	White (Ref)	0	0	.	.	.	.	.	.
**Construction Trade Work**	1	1	0.38394	0.25878	2.2012	0.1379	1.468	0.884	2.438
**Construction Trade Work**	0 (Ref)	0	0	.	.	.	.	.	.
**Exam Participant**	1	1	-0.76060	0.19774	14.7954	0.0001	0.467	0.317	0.689
**Exam Participant**	0 (Ref)	0	0	.	.	.	.	.	.
**Asbestosis, Silicosis or Cancer History**	1	1	0.63901	0.18193	12.3370	0.0004	1.895	1.326	2.706
**Asbestosis, Silicosis or Cancer History**	99[1]	1	-0.38302	0.51268	0.5581	0.4550	0.682	0.250	1.862
**Asbestosis, Silicosis or Cancer History**	0 (Ref)	0	0	.	.	.	.	.	.

Note: Gender not included in the model due to too few CRC cancer deaths among females.

[1] A value of 99 indicates missing.

Lung Cancer Mortality

Parameter	Value	DF	Parameter Estimate	Standard Error	Chi-Square	Pr > ChiSq	Hazard Ratio	95% Hazard Ratio Confidence Limits	
**Gender**	Female	1	0.06428	0.12523	0.2635	0.6077	1.066	0.834	1.363
**Gender**	Male (Ref)	0	0	.	.	.	.	.	.
**Race/Ethnicity**	Non-White	1	-0.02659	0.19485	0.0186	0.8915	0.974	0.665	1.427
**Race/Ethnicity**	White (Ref)	0	0	.	.	.	.	.	.
**Construction Trade Work**	1	1	0.77759	0.14850	27.4198	<.0001	2.176	1.627	2.911
**Construction Trade Work**	0 (Ref)	0	0	.	.	.	.	.	.
**Exam Participant**	1	1	-0.43944	0.10915	16.2098	<.0001	0.644	0.520	0.798
**Exam Participant**	0 (Ref)	0	0	.	.	.	.	.	.
**Asbestosis, Silicosis or Cancer History**	1	1	0.21602	0.09782	4.8766	0.0272	1.241	1.025	1.503
**Asbestosis, Silicosis or Cancer History**	99[1]	1	-0.02343	0.20653	0.0129	0.9097	0.977	0.652	1.464
**Asbestosis, Silicosis or Cancer History**	0 (Ref)	0	0	.	.	.	.	.	.

[1] A value of 99 indicates missing.

All Respiratory Disease Mortality

Parameter	Value	DF	Parameter Estimate	Standard Error	Chi-Square	Pr > ChiSq	Hazard Ratio	95% Hazard Ratio Confidence Limits	
**Gender**	Female	1	-0.09250	0.17865	0.2681	0.6046	0.912	0.642	1.294
**Gender**	Male (Ref)	0	0	.	.	.	.	.	.
**Race/Ethnicity**	Non-White	1	-0.33217	0.12568	6.9852	0.0082	0.717	0.561	0.918
**Race/Ethnicity**	White (Ref)	0	0	.	.	.	.	.	.
**Exam Participant**	1	1	-0.31204	0.08752	12.7108	0.0004	0.732	0.617	0.869
**Exam Participant**	0 (Ref)	0	0	.	.	.	.	.	.
**Construction Trade Work**	1	1	0.45943	0.10444	19.3522	<.0001	1.583	1.290	1.943
**Construction Trade Work**	0 (Ref)	0	0	.	.	.	.	.	.
**Asbestosis, Silicosis or Cancer History**	1	1	-0.02681	0.07590	0.1248	0.7239	0.974	0.839	1.130
**Asbestosis, Silicosis or Cancer History**	99[1]	1	0.04734	0.15389	0.0946	0.7584	1.048	0.775	1.418
**Asbestosis, Silicosis or Cancer History**	0 (Ref)	0	0	.	.	.	.	.	.

[1] A value of 99 indicates missing.

COPD Mortality

Parameter	Value	DF	Parameter Estimate	Standard Error	Chi-Square	Pr > ChiSq	Hazard Ratio	95% Hazard Ratio Confidence Limits	
**Gender**	Female	1	0.10168	0.21023	0.2339	0.6286	1.107	0.733	1.672
**Gender**	Male (Ref)	0	0	.	.	.	.	.	.
**Race/Ethnicity**	Non-White	1	-0.26493	0.15651	2.8652	0.0905	0.767	0.565	1.043
**Race/Ethnicity**	White (Ref)	0	0	.	.	.	.	.	.
**Exam Participant**	1	1	-0.45864	0.10896	17.7181	<.0001	0.632	0.511	0.783
**Exam Participant**	0 (Ref)	0	0	.	.	.	.	.	.
**Construction Trade Work**	1	1	0.44554	0.13422	11.0187	0.0009	1.561	1.200	2.031
**Construction Trade Work**	0 (Ref)	0	0	.	.	.	.	.	.
**Asbestosis, Silicosis or Cancer History**	1	1	-0.07429	0.10151	0.5356	0.4643	0.928	0.761	1.133
**Asbestosis, Silicosis or Cancer History**	99[1]	1	0.09011	0.19559	0.2122	0.6450	1.094	0.746	1.606
**Asbestosis, Silicosis or Cancer History**	0 (Ref)	0	0	.	.	.	.	.	.

[1] A value of 99 indicates missing.

All Cardiovascular Disease Mortality

Parameter	Value	DF	Parameter Estimate	Standard Error	Chi-Square	Pr > ChiSq	Hazard Ratio	95% Hazard Ratio Confidence Limits	
**Gender**	Female	1	-0.71982	0.14822	23.5847	<.0001	0.487	0.364	0.651
**Gender**	Male (Ref)	0	0	.	.	.	.	.	.
**Race/Ethnicity**	Non-White	1	0.20471	0.06882	8.8472	0.0029	1.227	1.072	1.404
**Race/Ethnicity**	White (Ref)	0	0	.	.	.	.	.	.
**Exam Participant**	1	1	-0.35558	0.05868	36.7169	<.0001	0.701	0.625	0.786
**Exam Participant**	0 (Ref)	0	0	.	.	.	.	.	.
**Construction Trade Work**	1	1	0.32073	0.06845	21.9556	<.0001	1.378	1.205	1.576
**Construction Trade Work**	0 (Ref)	0	0	.	.	.	.	.	.
**Asbestosis, Silicosis or Cancer History**	1	1	-0.03094	0.05336	0.3362	0.5620	0.970	0.873	1.076
**Asbestosis, Silicosis or Cancer History**	99[1]	1	0.07586	0.10457	0.5262	0.4682	1.079	0.879	1.324
**Asbestosis, Silicosis or Cancer History**	0 (Ref)	0	0	.	.	.	.	.	.

[1] A value of 99 indicates missing.

Ischemic Heart Disease Mortality

Parameter	Value	DF	Parameter Estimate	Standard Error	Chi-Square	Pr > ChiSq	Hazard Ratio	95% Hazard Ratio Confidence Limits	
**Gender**	Female	1	-0.52354	0.19157	7.4686	0.0063	0.592	0.407	0.862
**Gender**	Male (Ref)	0	0	.	.	.	.	.	.
**Race/Ethnicity**	Non-White	1	-0.08759	0.10623	0.6798	0.4097	0.916	0.744	1.128
**Race/Ethnicity**	White (Ref)	0	0	.	.	.	.	.	.
**Exam Participant**	1	1	-0.37097	0.08222	20.3588	<.0001	0.690	0.587	0.811
**Exam Participant**	0 (Ref)	0	0	.	.	.	.	.	.
**Construction Trade Work**	1	1	0.33436	0.09695	11.8945	0.0006	1.397	1.155	1.689
**Construction Trade Work**	0 (Ref)	0	0	.	.	.	.	.	.
**Asbestosis, Silicosis or Cancer History**	1	1	-0.08884	0.07616	1.3606	0.2434	0.915	0.788	1.062
**Asbestosis, Silicosis or Cancer History**	99[1]	1	-0.20992	0.16454	1.6277	0.2020	0.811	0.587	1.119
**Asbestosis, Silicosis or Cancer History**	0 (Ref)	0	0	.	.	.	.	.	.

[1] A value of 99 indicates missing.

All Other Causes Mortality

Parameter	Value	DF	Parameter Estimate	Standard Error	Chi-Square	Pr > ChiSq	Hazard Ratio	95% Hazard Ratio Confidence Limits	
**Gender**	Female	1	-0.28860	0.11028	6.8484	0.0089	0.749	0.604	0.930
**Gender**	Male (Ref)	0	0	.	.	.	.	.	.
**Race/Ethnicity**	Non-White	1	0.20189	0.06526	9.5721	0.0020	1.224	1.077	1.391
**Race/Ethnicity**	White (Ref)	0	0	.	.	.	.	.	.
**Exam Participant**	1	1	-0.26266	0.05923	19.6646	<.0001	0.769	0.685	0.864
**Exam Participant**	0 (Ref)	0	0	.	.	.	.	.	.
**Construction Trade Work**	1	1	0.13381	0.06249	4.5847	0.0323	1.143	1.011	1.292
**Construction Trade Work**	0 (Ref)	0	0	.	.	.	.	.	.
**Asbestosis, Silicosis or Cancer History**	1	1	0.07242	0.05231	1.9170	0.1662	1.075	0.970	1.191
**Asbestosis, Silicosis or Cancer History**	99[1]	1	-0.09152	0.10952	0.6984	0.4033	0.913	0.736	1.131
**Asbestosis, Silicosis or Cancer History**	0 (Ref)	0	0	.	.	.	.	.	.

[1] A value of 99 indicates missing.

Cox Proportional Hazard Model Results for All-Causes by BTMed Exam Participation Metrics (Manuscript Table 4)

Number of BTMed Exams vs Work History Only

Parameter	Value	DF	Parameter Estimate	Standard Error[2]	StdErr Ratio	Chi-Square	Pr > ChiSq	Hazard Ratio	95% Hazard Ratio Confidence Limits	
**Gender**	Female	1	-0.37835	0.06524	0.983	33.6294	<.0001	0.685	0.603	0.778
**Gender**	Male (Ref)	0	0	.	.	.	.	.	.	.
**Race/Ethnicity**	Non-White	1	0.12447	0.03790	1.016	10.7846	0.0010	1.133	1.051	1.220
**Race/Ethnicity**	White (Ref)	0	0	.	.	.	.	.	.	.
**Construction Trade Work**	1	1	0.32380	0.03619	0.992	80.0587	<.0001	1.382	1.288	1.484
**Construction Trade Work**	0 (Ref)	0	0	.	.	.	.	.	.	.
**One BTMed Exam**	1	1	-0.28886	0.03667	1.109	62.0350	<.0001	0.749	0.697	0.805
**Two BTMed Exams**	2	1	-0.34031	0.04065	1.075	70.0924	<.0001	0.712	0.657	0.771
**Three or More BTMed Exams**	3	1	-0.55703	0.05174	1.028	115.9226	<.0001	0.573	0.518	0.634
**Work History Only**	0 (Ref)	0	0	.	.	.	.	.	.	.
**Asbestosis, Silicosis or Cancer History**	1	1	0.12389	0.02828	1.011	19.1909	<.0001	1.132	1.071	1.196
**Asbestosis, Silicosis or Cancer History**	99[1]	1	0.01622	0.05534	0.947	0.0859	0.7695	1.016	0.912	1.133
**Asbestosis, Silicosis or Cancer History**	0 (Ref)	0	0	.	.	.	.	.	.	.

[1] A value of 99 indicates missing.

[2] Robust sandwich estimates of standard errors.

Number of Follow-up Exams among Exam Participants

Parameter	Value	DF	Parameter Estimate	Standard Error[1]	StdErr Ratio	Chi-Square	Pr > ChiSq	Hazard Ratio	95% Hazard Ratio Confidence Limits	
**Gender**	Female	1	-0.39831	0.07022	0.984	32.1798	<.0001	0.671	0.585	0.771
**Gender**	Male (Ref)	0	0	.	.	.	.	.	.	.
**Race/Ethnicity**	Non-White	1	0.09181	0.03925	0.974	5.4709	0.0193	1.096	1.015	1.184
**Race/Ethnicity**	White (Ref)	0	0	.	.	.	.	.	.	.
**Smoking Status**	Current Smoker	1	0.90094	0.03492	0.953	665.5849	<.0001	2.462	2.299	2.636
**Smoking Status**	Former Smoker	1	0.26327	0.02861	0.961	84.6537	<.0001	1.301	1.230	1.376
**Smoking Status**	Unknown	1	0.86546	0.09382	0.953	85.1035	<.0001	2.376	1.977	2.856
**Smoking Status**	Never Smoker (Ref)	0	0	.	.	.	.	.	.	.
**Hypertension History**	1	1	0.15640	0.02428	0.968	41.5076	<.0001	1.169	1.115	1.226
**Hypertension History**	0(Ref)	0	0	.	.	.	.	.	.	.
**Diabetes History**	1	1	0.40422	0.02694	0.957	225.1991	<.0001	1.498	1.421	1.579
**Diabetes History**	0 (Ref)	0	0	.	.	.	.	.	.	.
**Stroke History**	1	1	0.32945	0.04404	0.954	55.9697	<.0001	1.390	1.275	1.516
**Stroke History**	0 (Ref)	0	0	.	.	.	.	.	.	.
**Bronchitis/Emphysema History**	1	1	0.45422	0.02854	0.955	253.3027	<.0001	1.575	1.489	1.666
**Bronchitis/Emphysema History**	0 (Ref)	0	0	.	.	.	.	.	.	.
**Cancer History**	1	1	0.20949	0.02542	0.975	67.9108	<.0001	1.233	1.173	1.296
**Cancer History**	0 (Ref)	0	0	.	.	.	.	.	.	.
**Construction Trade Work**	1	1	0.25139	0.03880	0.970	41.9843	<.0001	1.286	1.192	1.387
**Construction Trade Work**	0 (Ref)	0	0	.	.	.	.	.	.	.
**Two BTMed Exams**	2	1	-0.00464	0.02662	0.961	0.0303	0.8618	0.995	0.945	1.049
**Three or More BTMed Exams**	3	1	-0.20259	0.04197	0.966	23.2953	<.0001	0.817	0.752	0.887
**Baseline Exam Only**	1 (Ref)	0	0	.	.	.	.	.	.	.

[1] Robust sandwich estimates of standard errors.

BTMed Exam Participants and ELCD Participants

Parameter	Value	DF	Parameter Estimate	Standard Error[1]	StdErr Ratio	Chi-Square	Pr > ChiSq	Hazard Ratio	95% Hazard Ratio Confidence Limits	
**Gender**	Female	1	-0.39858	0.07018	0.984	32.2548	<.0001	0.671	0.585	0.770
**Gender**	Male (Ref)	0	0	.	.	.	.	.	.	.
**Race/Ethnicity**	Non-White	1	0.08463	0.03927	0.974	4.6438	0.0312	1.088	1.008	1.175
**Race/Ethnicity**	White (Ref)	0	0	.	.	.	.	.	.	.
**Smoking Status**	Current Smoker	1	0.92753	0.03503	0.953	701.2627	<.0001	2.528	2.361	2.708
**Smoking Status**	Former Smoker	1	0.27790	0.02867	0.961	93.9419	<.0001	1.320	1.248	1.397
**Smoking Status**	Unknown	1	0.87247	0.09391	0.954	86.3170	<.0001	2.393	1.991	2.876
**Smoking Status**	Never Smoker (Ref)	0	0	.	.	.	.	.	.	.
**Hypertension History**	1	1	0.16202	0.02421	0.967	44.7922	<.0001	1.176	1.121	1.233
**Hypertension History**	0 (Ref)	0	0	.	.	.	.	.	.	.
**Diabetes History**	1	1	0.40942	0.02686	0.957	232.2844	<.0001	1.506	1.429	1.587
**Diabetes History**	0 (Ref)	0	0	.	.	.	.	.	.	.
**Stroke History**	1	1	0.32992	0.04398	0.954	56.2605	<.0001	1.391	1.276	1.516
**Stroke History**	0 (Ref)	0	0	.	.	.	.	.	.	.
**Bronchitis/Emphysema History**	1	1	0.45612	0.02853	0.955	255.6171	<.0001	1.578	1.492	1.669
**Bronchitis/Emphysema History**	0 (Ref)	0	0	.	.	.	.	.	.	.
**Cancer History**	1	1	0.21407	0.02533	0.976	71.4341	<.0001	1.239	1.179	1.302
**Cancer History**	0 (Ref)	0	0	.	.	.	.	.	.	.
**Construction Trade Work**	1	1	0.25069	0.03875	0.970	41.8465	<.0001	1.285	1.191	1.386
**Construction Trade Work**	0 (Ref)	0	0	.	.	.	.	.	.	.
**Exam and ELCD Participant**	2	1	-0.43032	0.06850	0.985	39.4677	<.0001	0.650	0.569	0.744
**Exam Participant Only**	1 (Ref)	0	0	.	.	.	.	.	.	.

[1] Robust sandwich estimates of standard errors.

Cox Model for All-cause Mortality by Time Since Completing the Work History Interview

Parameter	Value	DF	Parameter Estimate	Standard Error	Chi-Square	Pr > ChiSq	Hazard Ratio	95% Hazard Ratio Confidence Limits	
**Gender**	Female	1	-0.37860	0.06636	32.5474	<.0001	0.685	0.601	0.780
**Gender**	Male (Ref)	0	0	.	.	.	.	.	.
**Race/Ethnicity**	Non-White	1	0.12613	0.03730	11.4335	0.0007	1.134	1.054	1.220
**Race/Ethnicity**	White (Ref)	0	0	.	.	.	.	.	.
**Construction Trade Work**	1	1	0.31361	0.03645	74.0233	<.0001	1.368	1.274	1.470
**Construction Trade Work**	0 (Ref)	0	0	.	.	.	.	.	.
**Asbestosis, Silicosis or Cancer History**	1	1	0.14202	0.02795	25.8155	<.0001	1.153	1.091	1.218
**Asbestosis, Silicosis or Cancer History**	99[1]	1	0.00389	0.05841	0.0044	0.9469	1.004	0.895	1.126
**Asbestosis, Silicosis or Cancer History**	0 (Ref)	0	0	.	.	.	.	.	.
**Exam ≤ 12 Months Since Work History**	1	1	-0.33032	0.03215	105.5582	<.0001	0.719	0.675	0.765
**Exam >12 Months Since Work History**	2	1	-0.28507	0.05157	30.5615	<.0001	0.752	0.680	0.832
**Work History Only**	0 (Ref)	0	0	.	.	.	.	.	.

[1] A value of 99 indicates missing.
